# Glomerular C1q deposition and serum anti-C1q antibodies in anti-glomerular basement membrane disease

**DOI:** 10.1186/1471-2172-14-42

**Published:** 2013-09-21

**Authors:** Shui-yi Hu, Xiao-yu Jia, Xiao-wei Yang, Feng Yu, Zhao Cui, Ming-hui Zhao

**Affiliations:** 1 Renal Division, Department of Medicine, Peking University First Hospital, Beijing, China; 2 Institute of Nephrology, Peking University, Beijing, China; 3 Key Laboratory of Renal Disease, Ministry of Health of China; Key Laboratory of Chronic Kidney Disease, Prevention and Treatment, Ministry of Education, Beijing, China; 4 Peking-Tsinghua Center for Life Sciences, Beijing, China

**Keywords:** Anti-glomerular basement membrane disease, Complement, Classical pathway, C1q, Anti-C1q antibody

## Abstract

**Background:**

Anti-glomerular basement membrane (GBM) disease is a well-known antibody-induced autoimmune disease. A few patients have glomerular C1q deposition, but it is usually absent on renal histopathology. The role of C1q deposition in kidney injury is unclear. Recently, anti-C1q antibodies are demonstrated to be pathogenic in the target organ damage of many autoimmune diseases, by facilitating C1q deposition and enhancing complement activation via classical pathway. In the current study, we investigated the associations between anti-C1q antibodies in sera and C1q deposition in kidney of patients with anti-GBM disease.

**Results:**

It was shown that the severity of kidney injury was comparable between patients with and without C1q deposition, including the prevalence of oliguria/auria, the median percentage of crescents in glomeruli and the mean concentration of serum creatinine. Serum anti-C1q antibodies were detected in 15/25 (60%) patients with a low titer. The prevalence of C1q deposition in kidney was comparable between patients with and without serum anti-C1q antibodies (26.7% vs. 30.0%, p > 0.05). No association was found between anti-C1q antibodies and the severity of kidney injury.

**Conclusions:**

The classical pathway of complement may not play a pathogenic role in the kidney injury of human anti-GBM disease. Anti-C1q antibodies could be detected in more than half of patients, which need further investigations.

## Background

Anti-glomerular basement membrane (GBM) disease is a rare but severe autoimmune disorder which is clinically characterized by rapidly progressive glomerulonephritis with or without pulmonary hemorrhage. Anti-GBM antibodies have been proven to be pathogenic in the disease initiation [1,2]. The target autoantigen is on the non-collagenous domain of α3 chain of type IV collagen on GBM [α3(IV)NC1] [3]. On renal biopsy, linear deposition of anti-GBM IgG is shown along the glomerular capillary wall, which is always accompanied by linear or granular deposition of complement 3 (C3) [4]. This indicates that the complement system is activated and may involve in the pathogenesis of the disease.

Animal experiments demonstrated that complement activation via classical pathway is one of the major mechanisms for the glomerular injury of anti-GBM disease [5,6]. The pathway is triggered by the binding of C1q to anti-GBM IgG coupled with autoantigens, followed by the activation of C4, C2, C3, C5 and finally the formation of membrane attack complex, C5b-9. However, other animal experimental studies demonstrated the protective role for C1q against the renal inflammation using the accelerated nehprotoxic model [7]. In patients, C1q is seldom shown deposition along GBM on renal biopsy [4]. The circulating and urinary levels of C1q also present little correlation with the severity of kidney injury [8]. These make the pathogenic role of classical pathway activation doubtful in the disease.

The reason for the absence of C1q deposition is also unclear. We speculated it be due to the lack of autoantibody against C1q. Anti-C1q antibodies were first found in the circulation of patients with systemic lupus erythematosus [9] and later identified in many other autoimmune disorders [10-12]. Anti-GBM antibodies and anti-C1q antibodies have no cross reaction [13]. Recently, animal studies demonstrated that anti-C1q antibodies are pathogenic in the target organ damage, by facilitating the C1q deposition in tissues or cell membrane, and then enhancing the complement activation via classical pathway [14-16].

In the current study, we screened anti-C1q antibodies in sera of patients with anti-GBM disease. The correlation between the presence of serum anti-C1q antibodies and C1q deposition on kidney tissues was examined, together with other clinical and pathological data, with the aim to find the role of C1q deposition and serum anti-C1q antibodies in the disease.

## Results

### Demographic and clinical data of patients

Of the 25 patients, 22 (88%) were male and 3 were female, with the mean age at 31.8 ± 13.9 years. 12 (48%) patients presented with pulmonary hemorrhage. The mean level of serum creatinine on diagnosis was 991.3 ± 352.0 μmol/L. The mean level of serum anti-GBM antibodies was 88.6 ± 44.0 U/mL. 2 (8%) patients had positive ANCA, both with specificity for MPO. The levels of C3 and C4 in circulation were in normal range of all the patients.

**Figure 1:**
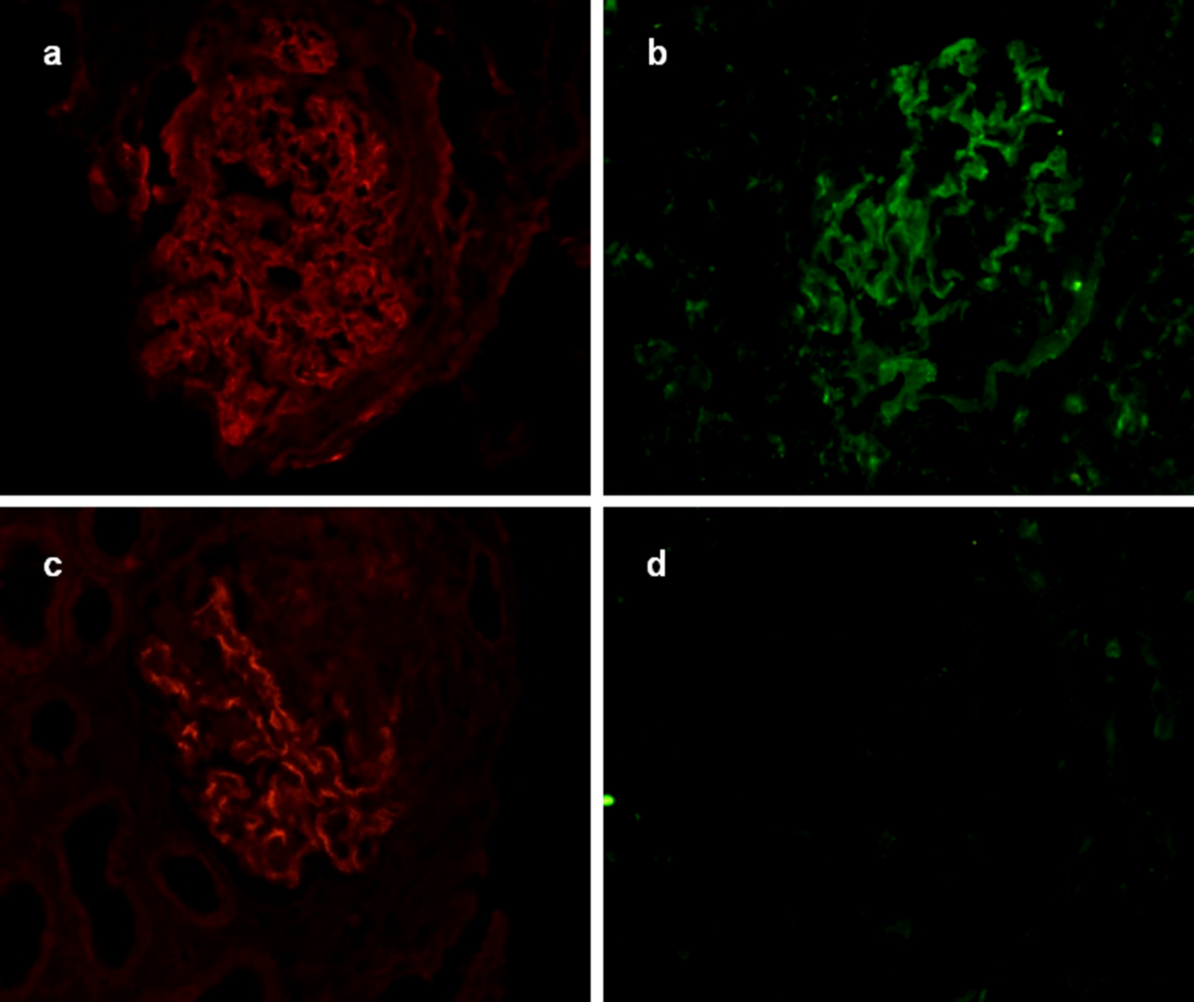
**C3c, C1q and C4d were detected on frozen renal tissues from patients by immunofluorescence (magnification × 400). a**: C3c granular deposition along the glomerular capillary wall; **b**: C1q granular deposition along the capillary wall; **c**: C4d granular deposition along the capillary wall. **d**: Negative control.

Renal biopsies showed linear staining of IgG, and linear or granular staining of C3c and C4d along GBM in all the 25 patients (Figure 1). Crescent formation was shown in the glomeruli with the range from 29% to 100%. 24 (96%) patients were diagnosed as crescentic glomerulonephritis (over 50% of the glomeruli had larger crescents). The percentage of crescents in glomeruli was positively correlated with the concentration of serum creatinine at presentation (r = 0.48, P = 0.016).

### The clinical and histopathological manifestations of patients with C1q deposition on GBM

The clinical features of the 7 patients with C1q deposition along GBM were listed in Table 1. Among them, 6 were male and one was female, with a mean age of 43.7 ± 11.8 years. 4 (57.1%) patients presented with lung hemorrhage. The mean level of serum creatinine on diagnosis was 914.3 ± 161.3 μmol/L. On renal biopsy, the mean percentage of crescent formation in the glomeruli was 83.3 ± 25.5%, with 6 patients diagnosed as crescentic glomerulonephritis and the rest one had crescents in 29% of the glomeruli. At the end of one year after treatment, all the 7 patients progressed to end stage renal disease (ESRD) and 4 (57.1%) patients died.

**Table 1:** Comparison of clinical and pathological data of patients with anti-GBM disease with and without glomerular C1q deposition

	**With C1q deposition (n = 7)**	**Without C1q deposition (n = 18)**	**p**
Age (years)	43.7 ± 11.8	27.2 ± 11.9	**0.005**
Gender (male/female)	6/1	16/2	1.000
Hydrocarbon exposure	1/7 (14.3%)	2/16 (12.5%)	1.000
Prodromal infection	2/7 (28.6%)	8/18 (44.4%)	0.653
Smoker	3/7 (42.9%)	8/18 (44.4%)	1.000
Hemoptysis	4/7 (57.1%)	8/18 (44.4%)	0.673
Oliguria/anuria	4/7 (57.1%)	10/18 (55.6%)	1.000
Gross hematuria	6/7 (85.7%)	6/18 (33.3%)	**0.03**
Nephrotic syndrome	2/3 (66.7%)	4/8 (50%)	1.000
Serum creatinine (μmol/L)	914.3 ± 161.3	1021.3 ± 402.9	0.353
Interval from onset (days)	48.6 ± 40.0	48.5 ± 33.3	0.998
Level of anti-GBM antibodies (U/mL)	88.0 ± 49.9	88.8 ± 43.0	0.969
ANCA positive	1/7 (14.3%)	1/18 (5.6%)	0.490
Hemoglobin (g/L)	89.6 ± 25.1	78.2 ± 29.5	0.377
Percentage of crescents in glomeruli (%)	91.0 (77.0-100.0)	100 (88.3-100.0)	0.386
Cellular crescents (%)	75.0 (50.0-100.0)	75.0(24.5-89.5)	0.389
Fibrocellular crescents (%)	25.0 (0–50)	25.0 (10.5-70.0)	0.336
Fibrotic crescents (%)	0	0	0.961
Anti-C1q antibody positive	4/7 (57.1%)	11/18 (61.1%)	1.000
Level of anti-C1q antibodies (relative OD value)	0.210 ± 0.05	0.245 ± 0.06	0.301
Renal survival (1 year)	0/7 (0)	2/18 (11.1%)	1.000
Patient survival (1 year)	3/7 (42.9%)	15/18 (83.3%)	0.066

*GBM* Glomerular basement membrane, *ANCA* Anti-neutrophil cytoplasmic antibodies.

The clinical features of patients with C1q deposition along GBM were compared with those without C1q deposition. It was shown that the patients with C1q deposition were older (43.7 ± 11.8 vs. 27.2 ± 11.9 years, p = 0.005) and presented with higher incidence of gross hematuria (85.7% vs. 33.3%, p = 0.03). However, the severity of kidney injury was comparable between patients with and without C1q deposition. The prevalence of oliguria/auria was comparable between them (57.1% vs. 55.6%, p > 0.05). The mean concentration of serum creatinine was equivalent between groups (914.3 ± 161.3 vs. 1021.3 ± 402.9 μmol/L, p > 0.05). On renal biopsy, the median percentage of crescents in glomeruli was also comparable between patients with and without C1q deposition (91.0(77.0-100.0) vs. 100(88.3-100.0)%, p > 0.05). No significance difference was observed between the two groups in gender, serum level of anti-GBM antibody, positive ANCA, lung hemorrhage, or the renal and patient outcomes (Table 1).

### The prevalence of anti-C1q antibodies

The cut-off value (relative OD value) of anti-C1q antibodies was 0.174. Sera from 15/25 (60%) patients were positive of anti-C1q antibodies in the circulation. The level of these autoantibodies ranged from 0.178 to 0.338, with a mean level of 0.236 ± 0.055.

### The association between the presence of anti-C1q antibodies and the clinical manifestations of patients

The clinical features of the 15 patients with positive serum anti-C1q antibodies were listed in Table 2. Of the 15 patients, 13 were male and 2 were female. The average age on diagnosis was 34.0 ± 16.0 years old. 6 (40%) patients had hemoptysis. 2 patients were ANCA positive. The mean level of serum creatinine on diagnosis was 1016.2 ± 340.0 μmol/L. On renal biopsy, the median percentage of crescents in glomeruli was 100.0(84.5-100.0)%, with 14 patients diagnosed as crescentic glomerulonephritis. At the end of one year follow up, one patient had his renal function recovered, the other 14 patients progressed to ESRD, and 5 patients died.

**Table 2:** Comparison of clinical and pathological data of patients with anti-GBM disease with and without serum anti-C1q antibodies

	**Anti-C1q antibody positive (n = 15)**	**Anti-C1q antibody negative (n = 10)**	**P**
Age (years)	34.0 ± 16.0	28.5 ± 9.8	0.342
Gender (male/female)	13/2	9/1	1.000
Hydrocarbon exposure	2/15 (13.3%)	1/10 (10%)	1.000
Prodromal infection	5/15 (33.3%)	5/10 (50%)	0.678
Smoker	6/15 (40%)	5/10 (50%)	1.000
Hemoptysis	6/15 (40%)	6/10 (60%)	0.428
Oliguria/anuria	8/15 (53.3%)	6/10 (60%)	1.000
Gross hematuria	6/15 (40%)	6/10 (60%)	0.428
Nephrotic syndrome	4/7 (57.1%)	2/4 (50%)	1.000
Serum creatinine (μmol/L)	1016.2 ± 340.0	954.1 ± 384.7	0.675
Interval from onset (days)	50.4 ± 33.4	46.0 ± 36.8	0.766
Level of anti-GBM antibodies (U/mL)	88.2 ± 49.0	89.2 ± 36.9	0.957
ANCA positive	2/15 (13.3%)	0/10 (0)	0.500
Hemoglobin (g/L)	78.2 ± 24.3	86.1 ± 34.3	0.506
Percentage of crescents in glomeruli (%)	100.0 (90.0-100.0)	88.5 (80.8-100.0)	0.210
Cellular crescents (%)	85.5 (24.8-100.0)	75.0 (29.5-83.3)	0.462
Fibrocellular crescents (%)	14.5 (0–50.0)	25.0 (16.8-68.0)	0.360
Fibrotic crescents (%)	0 (0–2.3)	0	0.443
Positive C1q deposit in the kidney	4/15 (26.7%)	3/10 (30%)	1.000
Renal survival (1 year)	1/15 (6.7%)	1/10 (10%)	1.000
Patient survival (1 year)	10/15 (66.7%)	8/10 (80%)	0.659

*GBM* Glomerular basement membrane, *ANCA* Anti-neutrophil cytoplasmic antibodies.

Glomerular C1q deposition was compared between patients with and without anti-C1q antibodies. Of the 15 patients with positive anti-C1q antibodies, 4 (26.7%) patients had glomerular C1q deposition. Of the 10 patients without serum anti-C1q antibodies, 3 (30%) patients had C1q deposition. The prevalence of glomerular C1q deposition was comparable between patients with and without anti-C1q antibodies (26.7% vs. 30%, p > 0.05).

The association between the presence of anti-C1q antibodies and the severity of kidney injury was further investigated. Of the 15 patients with positive anti-C1q antibodies, 8 (53.3%) patients had oliguria/auria. Of the 10 patients without anti-C1q antibodies, 6 (60%) patients had oliguria/auria. The prevalence of oliguria/auria was comparable between them (p > 0.05). Similarly, the mean concentration of serum creatinine was equivalent between patients with and without anti-C1q antibodies (1016.2 ± 340.0 vs. 954.1 ± 384.7 μmol/L, p > 0.05). On renal biopsy, the median percentage of crescents in glomeruli was also comparable between groups (100.0(90.0-100.0) vs. 88.5(80.8-100.0)%, p > 0.05). There was no significant difference between the two groups in gender, age, hemoptysis, anti-GBM antibody levels, ANCA prevalence, or the renal and patient outcomes (p > 0.05) (Table 2).

The levels of anti-C1q antibodies were all in the low range. No significant association was found between the level of anti-C1q antibodies and the clinical or pathological features (p > 0.05).

## Discussion

In the present study, 25 patients were investigated with renal biopsy-proven anti-GBM disease. They all had linear IgG deposition accompanied by C3 along GBM on the kidney tissue. This provides evidence that the complement system was activated in the glomeruli. In the current study, we also demonstrated the staining of C4d along GBM in all patients. This indicates the activation of classical pathway. However, on renal biopsy, C1q deposition is seldom shown in patients [4]. This makes the role of classical complement activation controversial in the kidney injury of anti-GBM disease.

In nephrotoxic nephritis (NTN) animal models, the role of complement activation via classical pathway has been studied using the C1q or C3 deficient mice [5-7]. In C1q knockout mice, C3 deposition caused by the induction of heterologous anti-GBM antibodies is attenuated, in comparison with the wild type mice [5,6]. This indicates a pathogenic role in the mechanism of glomerular damage. However, in other studies, a protective role for C1q against renal inflammation is revealed [7]. One explanation for these disparate findings is the two phases, the heterologous phase and the autologous phase, in the development of kidney injure in this model. The classical pathway of complement activation may exhibit different roles in different phases [17]. Another explanation may be the difference between the immune complex mediated glomerulonephritis in animal models and the antibody dependent cell cytotoxicity mechanism in human patients. Thus the mouse models are not reflecting human anti-GBM disease exactly and are of limited value.

In the current study, we analyzed the clinical and pathological data and outcomes of the patients with anti-GBM disease and compared the patients with and without glomerular C1q deposition in kidney. No association was identified between glomerular C1q deposition and the severity of renal injury or the outcomes of patients. Neither the percentage of crescent in glomeruli nor the level of serum creatinine was correlated with glomerular C1q deposition. These findings indicate that the classical complement activation may not play a major pathogenic role in the mechanism of glomerular damage in human anti-GBM disease. It is consistent with our previous study, in which we detected the level of circulating and urinary C1q and found little correlation with the severity of kidney injury [8]. The present study is limited due to the small sample size. The identification from multiple centers is needed in the future.

Both the classical and lectin pathways go through the activation of C4. C4d deposition was demonstrated on GBM in the glomeruli of our patients. The level of circulating and urinary mannose-binding lectin (MBL) was detected in our previous study, which had little correlation with the severity of kidney injury [8]. Recently, we examined the MBL in the kidneys of our patients and found that MBL deposited diffusively on the GBM and mesangial area and did not co-localize with C5b-9. However, the C1q deposit was stained in a linear pattern on GBM and co-localized well with C5b-9. These findings indicate that the complement cascade is activated through the classical pathway, whereas it does not show a direct pathogenic role to the kidney injury in human anti-GBM disease.

Animal studies using C4 knockout mice, which lack a functional classical and lectin pathway, provided strong evidence for the involvement of alternative pathway in the complement activation and development of anti-GBM disease [5,6]. After the induction of heterogeneous anti-GBM antibodies, C4^-/-^ mice developed albuminuria similar to that observed in wild type mice. We speculated that both the classical pathway and alternative pathway participate in the complement activation in human anti-GBM disease. The alternative pathway may play more pathogenic role in the kidney injury. The complement might be activated firstly via the classical pathway and generate the inflammatory molecules, such as C3a, C5a and C5b-9, which may be further amplified by the alternative pathway and be crucial in the effector phase of kidney injury [18-20]. These need further investigations.

The reasons for the absence of C1q deposition in routine direct immunofluorescence on renal biopsy are unclear. Anti-C1q antibodies have been demonstrated to facilitate the deposition of C1q in target organ and on cell surface [14,15]. In patients with lupus nephritis, the serum anti-C1q antibody levels are much higher in patients with C1q deposition in the kidney than those without C1q deposit [21]. Coremans *et al.* have also detected positive anti-C1q antibodies in less than 50% of patients with anti-GBM disease [13]. In the present study, we found 60% of patients having anti-C1q antibodies, whereas the deposition of C1q in glomeruli was not more frequently shown in the kidney. There may be two reasons for it. Firstly, the circulating anti-C1q antibodies were mostly in a lower level, which makes them less effective in facilitating the deposit of C1q. In SLE, the patients with lupus nephritis present much higher titers of anti-C1q antibodies than those without kidney injury. The higher titer of anti-C1q antibodies is also an important predictor for the renal flares [22,23]. Although we did detect the presentation of anti-C1q antibodies in the patients with anti-GBM disease, they were all in a lower level as they were in other autoimmune disease [12]. The lower titers may prevent the role of anti-C1q antibodies. Secondly, anti-C1q antibodies may help the autologous C1q deposit in healthy mice, but induce overt renal damage only in the context of glomerular immune complex disease [14-16]. As an organ-specific autoimmune disease, circulating immune complex does not play an important role in the pathogenesis of anti-GBM disease. The target organs are much prone to be damaged by the humoral and/or cellular mechanisms locally. There might be other explanation for the absence of glomerular C1q deposition. Unlike C3d and C4d, C1q does not bind covalently to its ligands, which results in its short half-life time *in vivo* and easy to be cleared by macrophages [24].

## Conclusions

The classical pathway of complement may not play a pathogenic role in the development of kidney injury of human anti-GBM disease. Serum anti-C1q antibodies could be detected in more than half of patients, which needs further investigations.

## Methods

### Patients and sera

Between 1996 and 2008, 25 patients with renal biopsy-proven anti-GBM disease were hospitalized in Peking University First Hospital. Seven patients, with anti-GBM IgG linear deposition and C3 and C1q linear or granular deposition along GBM by direct immunofluorescence, were used as study group. The other 18 patients, randomly selected from all the patients in the same period, who had anti-GBM IgG and C3 deposition along GBM, but no C1q deposition, were used as control group. Patients with secondary anti-GBM disease or with other coexisting renal diseases were excluded.

Clinical and pathological parameters were collected from medical records at the time of presentation and during follow up. Sera samples were collected at the day of renal biopsy before the immunosuppressive treatments and stored at -20°C until use. The research was in compliance of the Declaration of Helsinki and approved by the ethics committee of the Peking University First Hospital. Written informed consent was obtained from each participant.

### Detection of anti-GBM antibodies and ANCA

Sera from all patients were screened at presentation before the initiation of immunosuppressive treatment. Anti-GBM assays were performed by enzyme-linked immunosorbent assay (ELISA) using purified bovine α(IV)NC1 as solid phase antigen (EUROIMMUN, Lübeck, Germany), with confirmation of antibody specificity by ELISA against recombinant human α3(IV)NC1. Anti-neutrophil cytoplasmic antibody (ANCA) assays were performed by indirect immunofluorescence (EUROIMMUN, Lübeck, Germany) using ethanol-fixed human neutrophils. Antigen-specific ELISA was performed against purified myeloperoxidase (MPO) and proteinase 3 (PR3).

### Renal histopathology

Renal biopsy was performed at the time of diagnosis. Renal specimens were evaluated using direct immunofluorescence, light and electron microscopy and were forwarded to two pathologists. Both pathologists examined the biopsies separately, blinded to each other and the patients’ data.

For direct immunofluorescence, frozen sections were examined by a fluorescent microscope (Nikon, Tokyo, Japan) after staining with fluorescein isothiocyanate-conjugated antibodies specific for human IgG, IgM, IgA, C1q, fibrinogen and albumin, and tetramethylrhodamine isothiocyanate-labeled antibodies for C4d and C3c (Dako, Copenhagen, Denmark). For light microscopy, paraffin sections were stained with haematoxylin and eosin, periodic acid-schiff, periodic acid-silver methenamine and Masson’s trichrome. For electron microscopy, biopsy materials were fixed in glutaraldehyde, postfixed in osmium tetroxide, dehydrated in graded acetone and embedded in Epon 812 resin. Ultrathin sections were stained with uranyl acetate and lead citrate, and examined by a transmission electron microscope JEM-1230 (JEOL, Tokyo, Japan).

### Detection of anti-C1q antibodies

Detection of anti-C1q antibodies were performed by ELISA as previously described [25,26]. The positive control was the serum of one patient with lupus nephritis identified with positive anti-C1q antibodies and the negative control were the sera from healthy blood donors. Purified normal human C1q (Sigma, St Louis, MO, USA) diluted at 5 μg/mL in 0.05 M bicarbonate buffer (pH 9.6) was coated onto the wells of one-half of a polystyrene microtiter plate (Costar, Mankato, MN, USA) overnight at 4°C. The wells in the other half were coated with bovine serum albumin (BSA) diluted 5 μg/mL in 0.05 M bicarbonate buffer as antigen-free wells for exclusion of non-specific bindings. The volumes of each well for all steps were 100 μL. All incubations were carried out at 37°C for 1 hour and the plates were washed three times between every step with 0.01 M phosphate-buffered saline containing 0.1% Tween 20 (PBST). The plates were then blocked for 1 hour at 37°C with 1% (10 mg/ml) BSA in PBST. Sera were diluted to 1:50 in PBST/0.5 M NaCl and then added in duplicate to both antigen-coated and antigen-free wells. Each plate contained a blank, negative and positive control. The wells were then incubated with 1:5000 diluted alkaline phosphatase-conjugated goat anti-human IgG (Fc specific, Sigma, St Louis, MO, USA). The results were recorded as the net optical absorbance (average value of antigen-coated wells minus average value of antigen-free wells) at 405 nm (Bio-Rad 550, Tokyo, Japan). The absorbance value of positive controls should be around 1.5-2.0 in each assay. To make the results comparable between assays, the relative values for positive control and blank control were defined as 1.0 and 0.0, respectively. Autoantibody levels of the tested sera were expressed as relative values. Samples were considered positive if they exceeded the mean plus 2 standard deviations from 25 age- and gender-matched healthy blood donors.

### Statistical analysis

Differences of quantitative data were assessed using Student’s *t* test (for normally distributed data: serum creatinine, level of anti-GBM antibodies, hemoglobin and level of anti-C1q antibodies) or nonparametric test (for non-normally distributed data: percentage of crescents in glomeruli, cellular crescents, fibrocellular crescents, fibrotic crescents). Pearson’s correlation test was used to measure the correlation between two normally distributed variables. Spearman’s correlation test was used to measure the correlation between two non-normally distributed variables or one normally with one non-normally distributed variable. Univariate survival analysis was performed using the Cox regression model. All statistical analyses were two-tailed and P < 0.05 was considered as significant. Statistical software SPSS 18.0 (SPSS, Chicago, IL, USA) was employed for statistical analysis.

## Abbreviations

GBM: Glomerular basement membrane; ANCA: Anti-neutrophil cytoplasmic antibodies; MPO: Myeloperoxidase; PR3: Proteinase 3; MBL: Mannose-binding lectin.

## Competing interests

All authors declare that they have no competing interests.

## Authors’ contributions

SH carried out the immunoassays and drafted the manuscript. XJ contributed to collection of data. XY and FY helped to carry out the ELISA of anti-C1q antibodies detection and participated in the study design. ZC contributed to collection of data, and revised the critical content of manuscript. MZ conceived of the study and participated in its design and coordination and help to draft the manuscript. All authors read and approved the final manuscript.
